# The Impact of the ENDORSE Digital Weight Management Program on the Metabolic Profile of Children and Adolescents with Overweight and Obesity and on Food Parenting Practices

**DOI:** 10.3390/nu15071777

**Published:** 2023-04-05

**Authors:** Panagiota Pervanidou, Evi Chatzidaki, Nicolas C. Nicolaides, Antonis Voutetakis, Nektaria Polychronaki, Vassiliki Chioti, Rosa-Anna Kitani, Eleni Kyrkopoulou, Konstantia Zarkogianni, Eleftherios Kalafatis, Kostas Mitsis, Κonstantinos Perakis, Konstantina Nikita, Christina Kanaka-Gantenbein

**Affiliations:** 1 First Department of Pediatrics, School of Medicine, National and Kapodistrian University of Athens, Aghia Sophia Children’s Hospital, 11527 Athens, Greece; 2 Department of Pediatrics, School of Medicine, Democritus University of Thrace, 68100 Alexandroupolis, Greece; 3 Department of Economics, University of Piraeus, 18534 Pireas, Greece; 4 School of Electrical and Computer Engineering, National Technical University of Athens, 15780 Athens, Greece; 5 UBITECH, Big Data Science & Analytics Unit, 15231 Athens, Greece

**Keywords:** metabolic parameters, digital weight management program, child and adolescent obesity, food parenting practices

## Abstract

Childhood obesity is a serious public health problem worldwide. The ENDORSE platform is an innovative software ecosystem based on Artificial Intelligence which consists of mobile applications for parents and health professionals, activity trackers, and mobile games for children. This study explores the impact of the ENDORSE platform on metabolic parameters associated with pediatric obesity and on the food parenting practices of the participating mothers. Therefore, the metabolic parameters of the 45 children (mean age: 10.42 years, 53% girls, 58% pubertal, mean baseline BMI z-score 2.83) who completed the ENDORSE study were evaluated. The Comprehensive Feeding Practices Questionnaire was used for the assessment of food parenting practices. Furthermore, regression analysis was used to investigate possible associations between BMI z-score changes and changes in metabolic parameters and food parenting practices. Overall, there was a statistically significant reduction in glycated hemoglobin (mean change = −0.10, *p* = 0.013), SGOT (mean change = −1.84, *p* = 0.011), and SGPT (mean change = −2.95, *p* = 0.022). Emotional feeding/food as reward decreased (mean change −0.21, *p* = 0.007) and healthy eating guidance increased (mean change = 0.11, *p* = 0.051). Linear regression analysis revealed that BMI z-score change had a robust and significant correlation with important metabolic parameters: HOMA-IR change (beta coefficient = 3.60, *p*-value = 0.046), SGPT change (beta coefficient = 11.90, *p*-value = 0.037), and cortisol change (beta coefficient = 9.96, *p*-value = 0.008). Furthermore, healthy eating guidance change had a robust negative relationship with BMI z-score change (beta coefficient = −0.29, *p*-value = 0.007). Conclusions: The Endorse digital weight management program improved several metabolic parameters and food parenting practices.

## 1. Introduction

Childhood and adolescent obesity is a major public health issue with multiple biological and psychosocial risk factors and consequences [1,2,3]. Although the worldwide prevalence of childhood obesity tends to stabilize or even decrease, the prevalence of severe obesity (i.e., BMI ≥ 120% of the 95th centile) continues to rise, along with associated comorbidities including prediabetes, hypertension, and dyslipidemia [3,4,5,6]. According to Skinner et al., the greater the severity of obesity, the higher the risk of a low HDL cholesterol level, high systolic and diastolic pressure, high triglycerides, and glycated hemoglobin levels [5]. Furthermore, the recent COVID-19 pandemic has imposed changes in children’s lifestyles and behaviors leading to decreased physical activity, increased screen time, increased snacking, and so on, that can lead to excessive weight gain [7,8].

A reduction of 0.2 units of BMI z-score is considered a clinically significant weight reduction in pediatric weight management programs suggested by the US Preventive Services Task Force (USPSTF). To achieve this result, intensive multicomponent interventions are required, with a minimum of 26 contact hours and a duration of 3–6 months [9]. Furthermore, Reinehr et al. reported that a BMI-standard deviation score (BMI-SDS) reduction of 0.25 or more is necessary for managing hypertension, hypertriglyceridemia, and low HDL-cholesterol, whereas a BMI-SDS greater than 0.5 doubles the beneficial effect [10]. Another study by El-Medany et al. reported that a reduction of BMI-SDS >1, >1.2, or >0.7 led to a mean reduction of systolic blood pressure, LDL—cholesterol, and triglycerides, respectively [11]. Family-based pediatric lifestyle modification programs are considered first-line treatment options for childhood obesity [9,12,13,14,15]. These programs do not target weight only, but the overall health of children and the development of healthy habits in the family (i.e., healthy dietary patterns, physical activity, sedentary behavior, sleep). As highlighted by the American Psychological Association these interventions are not only provided to the child or adolescent but rather involve the parents and potentially other family members as active participants [12].

Parental feeding practices are goal-directed behaviors adopted by parents to directly influence their children’s eating behavior [16,17,18]. According to a recent meta-analysis of parental practices on child promotive and preventive food consumption, availability and parental modeling were strongly associated with both healthy and unhealthy food consumption in children [19]. Published data regarding the association between parental feeding practices and weight outcomes in children provided mixed results. While studies show that parents of children with overweight use monitoring and food restriction more often compared to parents of children with normal weight [20,21,22], evidence from a systematic review of 38 prospective studies found that restriction, pressure to eat, and monitoring were generally not associated with children’s weight over time [23]. Evidence from a family-based behavioral program for childhood obesity showed that modifications of parental feeding practices resulted in improvements in child standardized BMI [24].

Recently, digital weight management interventions have been developed and demonstrated promising results [25,26,27,28,29]. Especially in the COVID-19 era, digital interventions provide unique opportunities to increase access to weight management programs, since they require fewer human resources and time and are associated with less burden to individuals and health systems [25,30]. Most digital interventions report outcomes on anthropometric measures (weight, BMI, BMI z-score) [25,27,28], while only a few focus on cardiometabolic comorbidities such as prediabetes, high blood pressure, and dyslipidemia [31,32,33]. In the meta-analysis by Fowler et al., which included 32 randomized control trials of technology-based interventions for pediatric obesity, a small but significant effect on weight was found. However, treatment effects were significantly greater on outcomes for pilot interventions, interventions delivered to the child compared to parent-delivered interventions, and as child age increased and intervention duration decreased [25]. Evidence from systematic reviews exploring the role of parental involvement in the effectiveness of digital weight management programs is inconclusive [26,34]. A recent systematic review on the effectiveness of mobile health interventions targeting parents to treat childhood obesity concluded that only two out of the total nine intervention studies reported small differences between groups in the improvement of BMI z-score [34].

The ENDORSE platform is a novel and innovative digital platform that incorporates Artificial Intelligence, gamification, and biofeedback technologies capable of delivering tools and services that facilitate weight management of children and adolescents aged 6–14 years while incorporating the active involvement of caregivers and health professionals. The ENDORSE platform consisted of (a) a physical activity tracker that enabled physical activity monitoring and sleep tracking of children, (b) mobile applications for parents and health professionals thus enabling remote health monitoring and communication between parents and health professionals, (c) a mobile serious game designed to promote physical activity and healthy nutritional choices in children, and (d) the ENDORSE Recommendation System, i.e., an AI-based model able to produce personalized content to all end users. The usability and acceptability of the ENDORSE platform have been tested in 50 mother−child dyads who used the platform for 12 weeks. The usage of the ENDORSE platform resulted in clinically significant BMI z-score reduction and improvements in health behaviors including diet, physical activity, sedentary behavior, and sleep practices of the participating children [35].

The current study of the ENDORSE project presents the impact of the ENDORSE digital weight management program on selected metabolic parameters associated with pediatric obesity and on the food parenting practices of the participating mothers.

## 2. Materials and Methods

### 2.1. Participants and Setting

**Figure 1:**
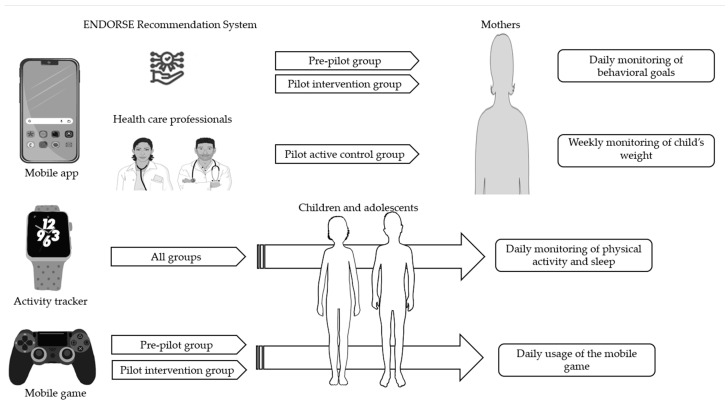
The components of the ENDORSE project consisted of mobile applications directed at both parents and health professionals and aimed to support self-monitoring by mothers. The ENDORSE Recommendation System generated personalized messages to mothers participating in the pre-pilot and pilot intervention groups, while in the pilot active control group, weekly personalized messages were sent by the healthcare professionals to mothers. Activity trackers were provided to all participating children to monitor physical activity and sleep, and an educational mobile game was provided to children participating in the pre-pilot and pilot intervention groups [35]. Parts of the figure were drawn using pictures from Servier Medical Art (smart.servier.com, accessed on 10 March 2023), provided by Servier, licensed under a Creative Commons Attribution 3.0 unported license and flaticon.com (accessed on 10 March 2023).

The ENDORSE pre−post intervention study included 50 children with overweight or obesity aged 6–14 years and was conducted in two phases from March 2021 to May 2022. The pre-pilot phase included 20 mothers with their children who used the initial version of the ENDORSE platform for 12 weeks (pre-pilot group). The pilot phase of the study included 30 mother−child dyads who used the platform for 12 weeks and consisted of two consecutive groups. The pilot active control group (n = 15) and the pilot intervention group (n = 15), as illustrated in Figure 1. The version of the ENDORSE platform used by the pilot active control group did not include the mobile game and the ENDORSE Recommendation System. A subsequent group of 15 mother−child dyads used the final complete version of the ENDORSE platform (pilot intervention group) that included all 4 components (mobile apps, activity trackers, mobile game, and ENDORSE Recommendation System). The overall dropout rate of the study was 10% (n = 5). The components of the ENDORSE platform are described in detail in Zarkogianni et al. [35].

Subjects were recruited from the Obesity outpatient Clinic of the First Department of Pediatrics of the Medical School of the National and Kapodistrian University, at the ‘Aghia Sophia’ Children’s Hospital in Athens, Greece, and were mostly residents of urban settings (i.e., n = 40, 90% residents of Athens). All mothers signed a consent form for themselves and their child’s participation in the study. The study was approved by the bioethics committee of ‘Aghia Sophia’ Children’s Hospital (protocol number: 4760, date of approval 10 March 2021). The theoretical framework of the ENDORSE study was self-determination theory (SDT) [36].

The present study focuses only on participants who completed the post-intervention assessment (n = 45, 90% of total participants) of the ENDORSE study. Based on the primary analysis of the ENDORSE study [35], which resulted in significant changes in BMI z-score, children were categorized into three groups according to BMI z-score change: group 1 with a clinically significant decrease in BMI z-score ≥ 0.2 according to the US Preventive Services Task Force (n = 21) [9]; group 2 with a decrease in/stable BMI z-score ≥ 0.00–0.20 (n = 16); and group 3 with an increase in BMI z-score > 0.00–0.25 (n = 8).

#### Inclusion/Exclusion Criteria

Children aged 6–14 years with a BMI >85th centile, which is the cut-off value for the definition of overweight according to the International Obesity Task Force (IOTF), were eligible to participate in the study [37]. Exclusion criteria for participation in the study were secondary causes of obesity, such as endocrine diseases (hypothyroidism, Cushing syndrome, growth hormone deficiency), known genetic syndromes linked to obesity (Down syndrome, Prader Willi syndrome, and so on), as well as serious neurodevelopmental conditions such as severe autism spectrum disorder (ASD) or severe attention-deficit hyperactivity disorder (ADHD) and psychiatric disorders.

### 2.2. Study Implementation

**Figure 2:**
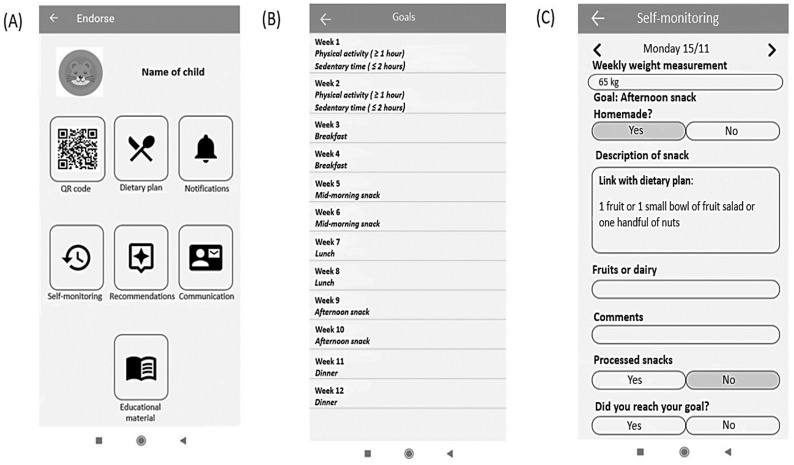
User interface of the parental version of the ENDORSE mobile application: (**A**) initial screen of the ENDORSE application, (**B**) summary of behavioral goals, and (**C**) example of self-monitoring of weight and health behavior goals [35].

In the ENDORSE study children were asked to monitor their physical activity (steps/day), and sleep via the activity tracker daily. They were also asked to use the educational mobile game daily. The ENDORSE parental mobile applications served multiple purposes, as mothers had access to their child’s personalized dietary plan, educational material, and several other functionalities, including communication with healthcare professionals via exchanging messages. Additionally, mothers were able to log in to the application the health behavior goals daily, and their child’s weight every week. All the components included in the ENDORSE parental version of mobile applications are illustrated in Figure 2 and aimed to support self-monitoring [35].

**Figure 3:**
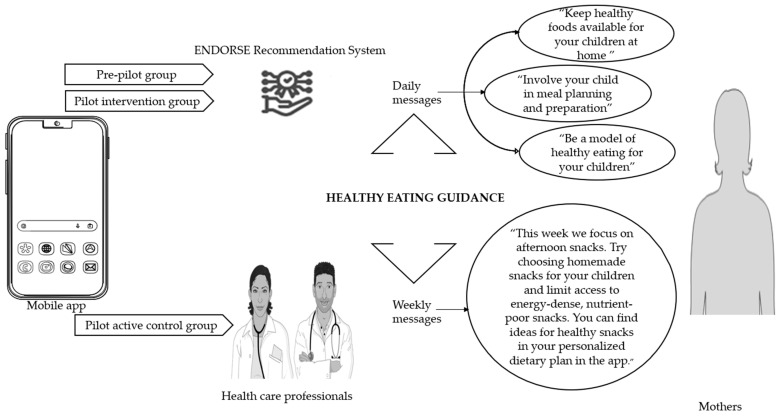
Healthy eating guidance was promoted through the ENDORSE digital platform [35]. Healthy eating guidance is a concept that includes the following five food parenting practices: encouragement of balance and variety in children’s diet, parental modeling of healthy eating, availability of healthy foods at home, child involvement in meal planning and preparation, and teaching about nutrition. It is based on the Greek version [38] of the Comprehensive Feeding Practices Questionnaire by Musher-Eizenman et Holub [39]. Parts of the figure were drawn using pictures from Servier Medical Art (smart.servier.com, accessed on 10 March 2023), provided by Servier, licensed under a Creative Commons Attribution 3.0 unported license and flaticon.com (accessed on 10 March 2023).

As illustrated in Figure 3 in the pre-pilot study and the pilot intervention group the ENDORSE Recommendation System generated daily messages targeting mothers that aimed to increase healthy eating guidance. The active control group received weekly messages from the clinical team via the application, that also aimed to increase healthy eating guidance. In the pilot intervention group, the weekly messages were not sent by the clinical team but were automatically generated and sent by the ENDORSE Recommendation System at the beginning of each week [35].

In the ENDORSE program emphasis was given to educating mothers about food parenting practices that promote healthy eating in children. In this direction, an educational booklet (printed and pdf version for the mobile application) specifically designed by the clinical team for the ENDORSE program, was given to mothers at the beginning of the study. The printed version of the educational material was given as an extra tool for teaching healthy nutrition to children at home. The material included a brief description of feeding practices according to the classification of Di Pasquale et Rivolta [16] through the framework of self-determination theory [36]. Emphasis was given to frequent family meals and children’s involvement in preparing meals, which are examples of relatedness-enhancing food parenting practices according to SDT, i.e., practices that pursue the goal of internalization of healthy eating behaviors by strengthening the child’s sense of relatedness to the parents as socializing agents in the feeding domain. Practices that provide structure and foster a child’s ability to conform to a healthy eating style, i.e., competence-enhancing food parenting practices, were also described (e.g., clear and consistent rules related to food availability and accessibility of healthy food at home, nutrition education, and parental modeling). Furthermore, emphasis was also given to autonomy-enhancing food parenting practices (i.e., guided choices, discussing and negotiating with the child’s food choices) which promote the child’s sense of autonomy in the feeding domain [16].

### 2.3. Measures

#### 2.3.1. Clinical Assessment

A full clinical assessment, including weight, height, BMI, blood pressure measurement, and pubertal staging was performed by a pediatric endocrinologist at baseline and the end of the study. According to their pubertal stage, children were characterized as prepubertal (Tanner stage 1) and pubertal (Tanner stage 2, 3, 4, and 5). Puberty was defined by the presence of breast Tanner stage ≥2 for girls and testicular volume of ≥4 mL in boys [40].

Height was measured to the nearest 1 mm with a stadiometer (Holtain Limited) with the child standing, wearing no shoes with his back against the wall and his body upright. Body weight was measured to the nearest 0.1 kg with the child dressed in light clothing using a mobile digital scale (Tefal Bodysignal). Body mass index (BMI) was calculated according to the equation: weight (kg)/height^2^ (m^2^). According to the age- and sex–specific definitions of the IOTF, overweight was defined as BMI > 85th centile, obesity as BMI > 95th centile, and severe obesity as BMI ≥ 120% of the 95th centile [4,37]. Each BMI was standardized by conversion to a z-score (BMI z-score) as defined by age and sex using the Centers for Disease Control and Prevention (CDC) growth charts [41]. Since BMI z-scores are known to be inaccurate at values greater than the 97th centile, an adjusted z-score was used for these children (n = 41, 91% of the whole sample) [42].

Waist circumference was measured with an elastic tape at the point halfway between the last palpable rib and the tip of the iliac crest with an accuracy of 0.1 cm. As a measure of adiposity, the waist-to-height ratio (WHR) was also calculated (WHR >0.5 is considered high, indicative of central obesity) [43]. Blood pressure (mmHg) was measured with an electronic blood pressure meter (Microlife Gentle) with the child seated, using an appropriate cuff. Two measurements were taken at each visit and the mean value was documented. According to the Endocrine Society, a blood pressure above the 90th centile for age, sex, and height is considered pre-hypertension [15,44].

#### 2.3.2. Laboratory Assessment

At the time of the clinical assessment, blood samples of the children were taken in the morning between 8 am and 9 am after overnight fasting at baseline and post-intervention. Twelve hours of fasting was recommended for the accuracy of blood test results.

Several biochemical and hormonal markers were assessed. Glucose was measured by a Cobas 6000 Clinical Chemistry Analyzer (Roche Diagnostics GmbH, Mannheim, Germany) using the enzymatic reference method with hexokinase (UV test). The normal range for glucose in our laboratory is 70–100 mg/dL, values above ≥ 100 mg/dL are considered markers of pre-diabetes [15,45]. Insulin was measured by Cobas e411 automated analyzer (Roche Diagnostics GmbH, Mannheim, Germany) using electrochemiluminescence immunoassay. The normal range for insulin in our laboratory is 2.6–25 μIU/mL. The homeostasis model assessment of insulin resistance (HOMA-IR) was calculated using the following formula: HOMA-IR = (insulin in mU/L × glucose in mg/dL)/405 [46]. According to van der Aa et al., levels of HOMA-IR >3.4 are indicative of insulin resistance in children and adolescents [47]. Glycated hemoglobin (HbA1c) was measured by Tosoh’s HLC-723G8 (Tosoh Europe N.V., Tessenderlo, Belgium), an automated glycohemoglobin analyzer (based on non-porous ion-exchange high-performance liquid chromatography). HbA1c is categorized as normal if <5.7%, while values between 5.7% and 6.1% are indicative of prediabetes, and values above 6.1% are indicative of diabetes [15,45]. Total cholesterol, LDL—cholesterol, HDL—cholesterol, and triglycerides were all measured by a Cobas 6000 Clinical Chemistry Analyzer (Roche Diagnostics GmbH, Mannheim, Germany) using the enzymatic colorimetric test. Normal values for total cholesterol are ≤170 mg/dL, for LDL—cholesterol ≤ 110 mg/dL, for HDL—cholesterol > 45 mg/dL, and for triglycerides ≤75 mg/dL for children aged 0–9 years, and TG ≤90 mg/dL for children aged 9–19 years [15,44].

Liver serum glutamic-oxaloacetic transaminase (SGOT) and serum glutamate pyruvate transaminase (SGPT) were measured by a Cobas 6000 Clinical Chemistry Analyzer (Roche Diagnostics GmbH, Mannheim, Germany) following the recommendation of the International Federation of Clinical Chemistry (IFCC) but were optimized for performance and stability. According to the Endocrine Society and Schwimmer et al., SGPT levels ≥25 U/L for boys and SGPT ≥22 U/L for girls are considered risk factors for fatty liver disease in children [15,48]. The stress biomarker ACTH was measured by an Immulite 2000 analyzer (Siemens Healthcare Diagnostics Products Ltd., Camberley, UK) using two-site chemiluminescent immunometric assays. Normal values in our laboratory are 9–52 pg/mL. Cortisol was measured by a Cobas e411 automated analyzer (Roche Diagnostics GmbH, Mannheim, Germany) using electrochemiluminescence immunoassay. Cortisol (μg/dL) exhibits different normal ranges according to age, sex, and Tanner stage.

#### 2.3.3. Nutritional Assessment

At baseline, a thorough nutritional assessment was performed by the team’s nutritionist. A diet history interview was used to collect qualitative information on usual dietary intake and preferences of main meals and intermediate snacks (type of foods, frequency, and usual amount), eating behavior, and parental feeding practices. Nutritional assessment information was used to design a nutritional intervention and an individualized meal plan for each child, which was provided to mothers through the ENDORSE mobile application. The Mediterranean dietary pattern, as described in the Greek National Dietary Guidelines for Infants, Children, and Adolescents, formed the basis of the individualized meal plan with macronutrient balance (15–20% protein, 30–35% fat, 50–55% carbohydrates) [49]. Energy requirements were calculated using the equations of the Institute of Medicine for use in pediatric populations with excess weight [50]. Energy requirements were adjusted according to weight maintenance or weight loss goal according to age and BMI categories based on the American Academy of Pediatrics (AAP) expert committee recommendations [13]. The dietary plan included a wide variety of foods relevant to each child’s preferences to encourage dietary adhesion.

#### 2.3.4. Assessment of Food Parenting Practices

Feeding practices were assessed pre-and post-intervention using the Comprehensive Feeding Practices Questionnaire by Musher-Eizenman et Holub [39], validated in Greece by Michou et al. [38]. Mothers completed the questionnaire via the mobile application. The Greek version of the questionnaire includes 42 questions that evaluate 6 feeding practices: (a) monitoring (i.e., parents keep track of child’s intake of less healthy foods), (b) child control (i.e., parents allow the child control of his/her eating behaviors and parent–child feeding interactions), (c) pressure to eat (i.e., parents exercise pressure on the child to consume more food at meals), (d) restriction (parents enforce strict limitations on the child’s access to foods or opportunities to consume these foods), (e) emotional feeding/food as reward (parents use food to control child’s negative emotions or use food as reward for child’s behavior), and (f) guidance for healthy eating [38]. Healthy eating guidance does not exist in the original questionnaire and includes the original subscales: encourage balance and variety (i.e., parents promote well-balanced food intake, including the consumption of varied foods and healthy food choices), modeling (i.e., parents actively demonstrate healthy eating for the child), environment (i.e., parents make healthy foods available at home), involvement (i.e., parents encourage child’s involvement in meal planning and preparation), and teaching about nutrition (i.e., parents use explicit didactic techniques to encourage the consumption of healthy foods). The questionnaire is a five-point Likert scale and for the first 13 questions, the possible answers are 1 = never, 2 = rarely, 3 = sometimes, 4 = very often, and 5 = always, while for the remaining 29 questions, the answers are: 1 = disagree, 2 = somewhat disagree, 3 = neither disagree nor agree, 4 = somewhat agree, and 5 = agree.

#### 2.3.5. Psychological Assessment

At baseline, a thorough psychological assessment was performed by the team’s psychologist, who assessed the eligibility of mothers and their children for their participation in the study based on clinical interviews with structured open-ended questions. By the time the mothers were interviewed by the psychologist, they had completed the following psychometric questionnaires via the application: PHQ-9: Greek version of the Patient Health Questionnaire-9 by Kroenke et al., which is a screening tool for detecting depressive symptoms in adults [51,52], and the EAT-26: Eating Attitudes Test—26 by Garner et al. [53] Greek version by Simos [54], which is a screening tool for detecting eating disorders in adults. Mothers also completed for their children the parental version of the Strengths and Difficulties Questionnaire (SDQ) by Goodman [55], Greek version by Bibou -Nakou et al. [56], which is a screening tool for detecting behavioral and emotional problems in children and adolescents.

#### 2.3.6. Sociodemographic Assessment of Mothers

The mothers completed a questionnaire via the application, which was specifically designed for the needs of the study and included questions about age, weight, height, nationality, marital status, level of education, income, and type of work. The body mass index of the mothers was calculated based on self-reported weight and height: BMI = weight (kg)/height^2^ (m^2^). Their categorization of BMI status into normal (BMI: 18.5 kg/m^2^ to 24.9 kg/m^2^), overweight (BMI: 25 kg/m^2^ to 29.9 kg/m^2,^), and obese (BMI ≥ 30 kg/m^2^) was done according to the definition of overweight and obesity of the World Health Organization [57].

### 2.4. Statistical Analysis

Descriptive statistics were used to assess baseline participant characteristics categorized according to a reduction in BMI z-score (means ± SD, median with 25th and 75th centiles, or as absolute values with percentages). The Chi-square test was used for categorical variables, One-Way ANOVA for normally distributed variables, and Kruskal−Wallis for non-normally distributed variables. Paired sample *t*-tests were used to assess pre- and post-intervention changes within each of the three groups for normally distributed variables and the Wilcoxon test for non-normally distributed variables. Bivariate regression analysis was used to assess possible associations between BMI z-score change and the change in several metabolic parameters (systolic and diastolic blood pressure, glucose, glycated hemoglobin, insulin, HOMA-IR, total cholesterol, LDL-cholesterol, HDL-cholesterol, triglycerides, SGPT, SGOT, cortisol, and ACTH). Parameters that were significantly associated in bivariate regression were entered into multivariate regression models with covariates sex, pubertal stage, age (in years), and BMI z-score at baseline. Bivariate analysis was also used for the investigation of any associations between change in food parenting practices (healthy eating guidance, child control, monitoring, emotional feeding/food as reward, pressure to eat, restriction) and change in BMI z-score. Parameters that were significantly associated in bivariate regression were entered into multivariate regression models with covariates being maternal education and marital status. A value of *p* < 0.05 was considered statistically significant. The analysis was performed with the STATA statistical program.

## 3. Results

### 3.1. Participant’s Characteristics

The demographic, clinical, and behavioral characteristics of children in the three groups are presented in Table 1. A total of 45 children (53% girls) with a mean age of 10.4 years were included in the analysis. The mean BMI z-score was 2.83. Children in group 1 had a higher baseline BMI z-score (mean BMI z-score: 3.18 versus 2.47 in group 2 and 2.36 in group 3), but the difference was not statistically significant. No statistical differences were identified between groups according to age, sex, and pubertal stage. Children in group 3 had a longer mean follow-up period of 5.5 months versus 4.5 months in the other 2 groups (*p* = 0.05).

The baseline metabolic characteristics of the participating children in the three groups are presented in Table 2. No statistical differences were identified between groups, except for a borderline significant difference in baseline plasma glucose (lower in group 2, *p* = 0.055). Overall, according to the guidelines published by the Endocrine Society for screening for comorbidities of pediatric obesity [15], 22% of the children met the criteria for prediabetes (HbA1c: 5.7–6.5% or glucose ≥100 mg/dL), 30% of the children had low HDL cholesterol (<45 mg/dL), 42% had high triglycerides (>75 mg/dL in children younger than 9 years or >90 mg/dL in children older than 9 years), while 57% of the children met criteria for screening of fatty liver disease (SGPT > 25 U/L in boys and SGPT > 22 U/L in girls). According to the definition of insulin resistance by van der Aa et al. [47], 73% of the children had levels of HOMA-IR above 3.4.

Table 3 shows baseline maternal characteristics. The children’s mothers had a mean age of 44 years and a mean BMI of 30.2 kg/m^2^. The majority were Greek (96%) and married (78%). Most mothers (60%) were primary and secondary school graduates and 78% were employed. Twenty percent of the mothers had depressive symptomatology, while 16% were at risk for eating disorders. Regarding feeding practices, mothers used monitoring, healthy eating guidance, and restriction frequently. Nine percent of the mothers raised concerns about their child’s emotional and behavioral health according to the Strengths and Difficulties Questionnaire. No statistically significant differences were observed between the groups.

### 3.2. Changes in Cardiometabolic Factors According to BMI z-Score Change

Table 4 shows the changes in cardiometabolic parameters of the children participating in the ENDORSE study according to BMI z-score reduction. Overall, there was a statistically significant reduction in glycated hemoglobin (mean change = −0.10, *p* = 0.013), SGOT (mean change = −1.84, *p* = 0.011), and SGPT (mean change = −2.95, *p* = 0.022). Between groups, there were statistically significant differences in changes of the following metabolic parameters: glycated hemoglobin (greater reduction in group 2, *p* = 0.026), total cholesterol (decrease in groups 1 and 2, increase in group 3, *p* = 0.009), LDL—cholesterol (decrease in group 1 and increase in groups 2 and 3, *p* = 0.003), and cortisol (reduction in group 1, increase in groups 2 and 3, *p* = 0.037).

### 3.3. Linear Regression Analysis between Change in BMI z-Score and Change in Metabolic Parameters of Children Participating in the ENDORSE Program

First, we tested in the bivariate analysis whether BMI z-score change was associated with 14 metabolic parameters (systolic and diastolic blood pressure, glucose, glycated hemoglobin, insulin, HOMA-IR, total cholesterol, LDL-cholesterol, HDL-cholesterol, triglycerides, SGPT, SGOT, cortisol, and ACTH). Insulin, HOMA-IR, SGPT, cortisol, and LDL-cholesterol were found to be significantly associated with BMI z-score change and were entered in multivariate regression models with covariates sex (a binary variable that takes the value one if the child is female and 0 if the child is male), pubertal stage at baseline (a binary variable that takes the value one if the child is pubertal at baseline and 0 if the child is prepubertal at baseline), age (in years), and BMI z-score at baseline. The results for HOMA-IR, SGPT, and cortisol remained significant and are presented in Table 5, Table 6 and Table 7. As illustrated in the tables, one unit increase in the BMI z-score change was correlated with a 3.6 unit increase in HOMA-IR change (*p* = 0.046), 11.90 units increase in SGPT change (*p* = 0.037), and 9.96 units increase in cortisol change (*p* = 0.008), respectively.

### 3.4. Food Parenting Practices and BMI z-Score Change

Table 8 shows changes in food parenting practices of the mothers participating in the ENDORSE program according to BMI z-score reduction. Overall, there was a statistically significant decrease in emotional feeding/food as reward (mean change −0.21, *p* = 0.007) and an increase in healthy eating guidance (mean change = 0.11, borderline significant at *p* = 0.051). Between the groups, there were statistically significant differences in healthy eating guidance (groups 1 and 2 increase versus decrease in group 3, *p* = 0.013) and child control (decrease in groups 1 and 2 versus increase in group 3, *p* = 0.037).

### 3.5. Linear Regression Analysis for Healthy Eating Guidance and BMI z-Score

First, we entered all six food parenting practices (healthy eating guidance, child control, monitoring, emotional feeding/food as reward, pressure to eat, and restriction) into a bivariate analysis. Only healthy eating guidance had a statistically significant impact on BMI z-score change and was entered in a multivariate regression model with the covariates being maternal education and marital status. The multivariate regression analysis results are presented in Table 9. Healthy eating guidance change has a negative relationship with BMI z-score change that is significant at the 1% level after adjusting for both maternal education (primary, secondary, or tertiary education) and marital status (married mothers versus unmarried mothers).

## 4. Discussion

Numerous weight loss studies for childhood and adolescent obesity with on-site visits have been published so far and have demonstrated improvements in several clinical and cardiometabolic parameters [10,11,58,59]. However, the evidence concerning digital interventions is scarce [31,32,33]. In the current study, we were able to demonstrate that the implementation of the ENDORSE digital platform aiming at weight management and parental food practice modification resulted in a statistically significant reduction in glycated hemoglobin (mean change = −0.10, *p* = 0.013), with the greatest reduction observed in the group with a stable/decrease BMI z-score (−0.24, *p* = 0.026), while no significant changes in glucose were observed. Moreover, a robust and significant correlation between BMI z-score change and HOMA-IR change (beta coefficient = 3.60, *p*-value = 0.046) was demonstrated upon the use of the ENDORSE platform. An earlier study using digital interventions by video games that encourage healthy behavior found no change in fasting insulin [32]. Many studies with on-site visits targeting lifestyle behaviors in children with overweight and obesity have demonstrated improvements in glucose and insulin sensitivity [10,59,60,61,62,63,64]. In a prospective study of 1388 children who are overweight following a 1-year lifestyle intervention, a BMI-SDS reduction of 0.25–0.5 was related to a decrease in HOMA-IR (−0.5 ± 0.3) [10].

In the current study, the group of children with clinically significant weight reduction (BMI z-score reduction ≥0.2) demonstrated the greatest reduction in LDL-cholesterol. while we found no significant changes in other lipids (triglycerides, HDL-cholesterol, total cholesterol). Another digital intervention using long-term telephone aftercare preceded by a summer camp and involving 71 children with obesity has demonstrated a significant improvement in the levels of cholesterol, triglycerides, and LDL-cholesterol one year after active intervention [31]. Many on-site studies have demonstrated significant changes in lipids after weight loss in children [10,11,58,59,60,62,63]. A recent meta-regression analysis concerning LDL-cholesterol included 46 studies to investigate which level of BMI-SDS change in lifestyle interventions is required to improve cardiometabolic outcomes in children with obesity (aged 4–19 years),. The authors concluded that, for a meaningful reduction in mean LDL-cholesterol, a BMI-SDS reduction >1.2 is required [11].

In all groups, concentrations of liver transaminases decreased significantly, i.e., SGPT (mean change −2.95, *p* = 0.022) and SGOT (mean change −1.84, *p* = 0.011). We also demonstrated a robust and significant correlation between BMI z-score change and changes in the level of liver transaminase SGPT (beta coefficient = 11.90, *p*-value = 0.037). Obesity is considered the most significant risk factor for nonalcoholic fatty liver disease (NAFLD) and effective weight loss intervention is the key to disease treatment and prevention of NAFLD progression [63,65]. In the ENDORSE program, all children were given an individualized meal plan based on the Mediterranean dietary pattern. The Mediterranean Diet improves hepatic steatosis, and insulin sensitivity in individuals with nonalcoholic fatty liver disease [66]. In a pilot study of 18 children with obesity (10.8 ± 1.6 years; 63% females; BMI z-score 3 ± 0.4), a mean reduction of BMI z-score of 0.30 was achieved after 16 weeks of combined physical activity and nutritional counseling, which led to a significant mean reduction in the liver transaminase SGPT [63].

The group with clinically significant weight reduction had the greatest reduction in cortisol (mean change −3.87, *p* = 0.037). We have also demonstrated a robust and significant relationship between BMI z-score change and serum cortisol change (beta coefficient = 9.96, *p*-value = 0.008). The association between cortisol levels and weight loss has been investigated in weight management trials in both adults [67] and children [68,69], with significant decreases in serum cortisol after weight loss. To our best knowledge, only one digital intervention has been published; a mobile health intervention with a randomized controlled trial design, including 41 children with excess weight. This study showed a significant decrease in serum cortisol after the intervention, although BMI-SDS did not decrease after 12 months of follow-up [70].

In the current study that investigated the use of the ENDORSE digital weight management platform, healthy eating guidance increased post-intervention (mean change = 0.11, *p* = 0.051). Furthermore, regression analysis revealed a robust and significant negative relationship between healthy eating guidance change and BMI z-score change (beta coefficient = −0.29, *p*-value = 0.007). Healthy eating guidance includes practices that encourage healthy eating in children (i.e., modeling, involvement, availability of healthy foods in the home environment, encouragement, and nutrition education) [16,17,18]. A recent meta-analysis of 12 web-based interventions for changing parental feeding practices targeting parents of children younger than 12 years found that only food availability/accessibility led to a significant improvement in weight management [71]. This meta-analysis, however, included mainly parents of younger children. In our study based on the use of the ENDORSE digital weight management and parental practices approach we have also found statistically significant differences in child control within groups (increase in child control in the group with a BMI z-score increase as compared to the other two groups). Child control includes practices such as allowing children to eat whatever they want or whenever they want. In recent food parenting practice classifications, child control is classified under unstructured/indulgent practices [17,18]. These unstructured practices are not well defined in the literature, but correspond to the indulgent feeding style that characterizes parents who make few demands on what or how much their children eat, but are very supportive and warm in their efforts to promote healthy eating [18,72]. Studies have shown that the indulgent feeding style is associated with increased weight in children [73,74].

In all groups, emotional feeding/food as reward decreased significantly after the intervention (mean change −0.21, *p* = 0.007). Evidence from prospective studies shows that food as reward, but not emotional feeding, was associated with higher weight over time, but the authors highlight the fact that higher quality studies are required to confirm this association [23]. According to Di Pasquale et Rivolta, food as reward or emotional feeding could be interpreted as an attempt of the parents to compensate for the absence of emotional support and coaching for their child and can be defined as relatedness-thwarting food parenting practices [16]. Emotional feeding is an important target for future weight management programs targeting parents because it is prospectively linked to emotional eating in children [75,76]. Interestingly, emotional eating is a behavior that is learned and not inherited according to twin studies, with a heritability of only 7% compared to other eating behaviors, with a strong genetic basis (63% for satiety responsiveness, 75% for food responsiveness, and 78% for food fussiness) [75,77,78]. The newly released guidelines of the American Academy of Pediatrics regarding the evaluation and treatment of children and adolescents with obesity [79], endorsed by the American Heart Association [80], highlight family and home environment factors as important influences on children’s appetitive behaviors and food preferences.

### Limitations

The use of the ENDORSE digital weight management and parental food practices approach was conducted under challenging conditions imposed by the application of public health measures to control the COVID-19 pandemic. This resulted in difficulty in considering a control group following the standard of care due to the restricted clinical visits. The size of the recruited sample has also been affected and led to a limited number of participants. Moreover, the clinical sample that included a high proportion of children with severe obesity (42%) and few subjects who are overweight (8%), makes the sample not representative of all children with excess weight. Other limitations of the study are the lack of a follow-up period and the fact that we did not investigate the role of other family members (i.e., fathers and siblings).

## 5. Conclusions

In conclusion, the implementation of the ENDORSE digital weight management program resulted in improvements in several important metabolic parameters and food parenting practices. The use of this novel digital-based ENDORSE platform in weight management and the promotion of healthy parental food practices adds new data on the importance of digital-based interventions to combat childhood obesity. Certainly, future large-scale cohorts in combination with a control group representing the standard care of children and adolescents with overweight and obesitye are needed to further support the findings of this study.

## Figures and Tables

**Table 1:** Characteristics of children according to BMI z-score group.

Characteristics	Total (n = 45)	Group 1 Decrease in BMI z-Score ≥0.2 (n = 21)	Group 2 Decrease in BMI z-Score ≥0.0 <−0.2 (n = 16)	Group 3 Increase in BMI z-Score >0.00–0.25 (n = 8)	* *p* Value for Differences between Groups
Mean Follow-up Duration (Baseline to the last visit, months)	4.71 (1.05)	4.53 (0.91)	4.52 (1.09)	5.52 (1.02)	0.050
Age (years)	10.42 (2.04)	10.27 (2.17)	10.64 (1.64)	10.41 (2.61)	0.867
Sex					0.349
Female	24 (53.3)	11 (52.4)	7 (43.8)	6 (75.0)	
Male	21 (46.7)	10 (47.6)	9 (56.3)	2 (25.0)	
Pubertal Stage					0.840
Prepubertal	19 (42.2)	9 (42.9)	6 (37.5)	4 (50.0)	
Pubertal	26 (57.8)	12 (57.1)	10 (62.5)	4 (50.0)	
Weight (kg)	69.42 (23.48)	72.39 (26.25)	66.41 (21.54)	67.65 (21.28)	0.734
Height (m)	1.49 (0.14)	1.49 (0.14)	1.49 (0.12)	1.48 (0.17)	0.993
BMI (kg/m^2^)	30.52 (6.46)	31.59 (6.85)	29.32 (6.62)	30.08 (5.21)	0.568
BMI z-score	2.83 (2.22, 3.97)	3.18 (2.67, 4.32)	2.47 (2.00, 3.03)	2.36 (1.95, 4.97)	0.109
Weight Status (IOTF) [4]					0.457
Overweight	4 (8.9)	2 (9.5)	1 (6.3)	1 (12.5)	
Simple Obesity	23 (51.1)	8 (38.1)	11 (68.8)	4 (50.0)	
Severe Obesity	18 (40.0)	11 (52.4)	4 (25.0)	3 (37.5)	
Waist-to-Height Ratio	0.63 (0.08)	0.64 (0.09)	0.60 (0.06)	0.64 (0.07)	0.176
Systolic BP (mmHg)	115.0 (108.0, 120.0)	115.0 (109.5, 120.0)	115.0 (104.5, 120.8)	115.0 (109.3, 116.5)	0.902
Diastolic BP (mmHg)	73.84 (8.40)	73.95 (9.72)	73.06 (6.82)	75.13 (8.41)	0.855

Values are expressed as mean (SD) or median (25th and 75th percentiles) for continuous variables and as absolute numbers (n) and frequencies (%) for categorical variables. ΒΜΙ: Body Mass Index, BP: Blood pressure. * Pearson Chi-square test, ANOVA, or Kruskal−Wallis as indicated.

**Table 2:** Baseline metabolic characteristics of children according to BMI z-score group.

Characteristics	Total (n = 45)	Group 1 Decrease in BMI z-Score ≥0.2 (n = 21)	Group 2 Decrease in BMI z-Score ≥0.0 <−0.2 (n = 16)	Group 3 Increase in BMI z-Score >0.00–0.25 (n = 8)	* *p* Value for Differences between Groups
Glucose (mg/dL)	90.02 (7.71)	92.76 (9.02)	86.69 (5.39)	89.43 (5.13)	0.055
Insulin (μUI/mL)	23.90 (17.80, 35.40)	30.55 (18.88, 39.33)	23.40 (17.80, 28.80)	22.15 (12.73, 32.68)	0.302
HOMA IR	5.60 (4.05, 7.68)	7.04 (4.28, 9.80)	4.62 (3.96, 6.07)	4.48 (2.19, 6.18)	0.110
HbA1c (%)	5.44 (0.28)	5.39 (0.30)	5.46 (0.27)	5.51 (0.26)	0.566
Cholesterol (mg/dL)	158.31 (25.72)	165.28 (26.19)	156.00 (22.67)	145.50 (27.65)	0.168
LDL Cholesterol (mg/dL)	88.77 (21.65)	94.35 (22.04)	85.25 (17.92)	81.88 (26.45)	0.284
HDL Cholesterol (mg/dL)	49.60 (11.10)	48.53 (11.13)	52.88 (11.89)	45.75 (8.55)	0.287
Triglycerides (mg/dL)	85.00 (60.25, 133.25)	104.00 (64.25, 162.25)	80.00 (53.00, 94.25)	84.00 (60.25, 120.50)	0.512
SGPT (U/L)	22.00 (15.50, 31.00)	25.00 (14.50, 35.50)	25.00 (17.25, 35.25)	16.00 (11.75, 19.00)	0.071
SGOT (U/L)	23.00 (17.00, 26.50)	22.00 (17.00, 26.00)	24.50 (17.25, 29.25)	23.00 (16.00, 24.75)	0.627
Cortisol (μg/dL)	13.54 (5.12)	15.10 (5.60)	12.43 (3.72)	11.45 (5.55)	0.156
ACTH (pg/mL)	23.15 (15.35, 23.95)	24.10 (12.80, 40.15)	20.80 (16.10, 25.00)	31.10 (14.90, 39.20)	0.713

Values are expressed as mean (SD) or median (25th and 75th percentiles). HOMA IR: Homeostatic Model Assessment of Insulin Resistance, HbA1c: Glycated hemoglobin, LDL: LDL-cholesterol: Low-density lipoprotein cholesterol, HDL: High-density lipoprotein cholesterol, SGPT: Serum Glutamic Pyruvic Transaminase, SGOT: Serum Glutamyl Oxaloacetic Transaminase, ACTH: Adrenocorticotropic hormone. * One-Way ANOVA or Kruskal−Wallis as indicated.

**Table 3:** Baseline parental (maternal) characteristics according to their children’s BMI z-score group.

Characteristics	Total (n = 45)	Group 1 Decrease in BMI z-Score ≥0.2 (n = 21)	Group 2 Decrease in BMI z-Score ≥0.0 <−0.2 (n = 16)	Group 3 Increase in BMI z-Score >0.00–0.25 (n = 8)	* *p* Value for Differences between Groups
Maternal age (years)	43.89 (5.40)	44.19 (5.47)	43.75 (3.32)	43.38 (8.55)	0.931
Maternal BMI (kg/m^2^)	30.18 (6.15)	30.74 (6.15)	30.60 (6.40)	27.85 (5.84)	0.509
Mothers of Greek origin	43 (95.6)	20 (95.2)	16 (100.0)	7 (87.5)	0.373
Married mothers	35 (77.8)	18 (85.7)	11 (68.8)	6 (75.0)	0.459
Maternal education					0.881
Primary	4 (8.9)	2 (9.5)	1 (6.3)	1 (12.5)	
Secondary	23 (51.3)	12 (57.1)	8 (50.0)	3 (37.5)	
Tertiary	18 (40.0)	7 (33.3)	7 (43.8)	4 (50.0)	
Annual income					0.823
Less than 10,000 euros/year	11 (24.4)	7 (33.3)	3 (18.8)	1 (12.5)	
10,000–20,000 euros/year	10 (22.2)	5 (23.8)	3 (18.8)	2 (25)	
More than 20,000 euros/year	4 (8.9)	2 (9.5)	1 (6.3)	1 (12.5)	
Omission	20 (44.4)	7 (33.3)	9 (56.3)	4 (50)	
Satisfaction from income					0.490
(5-point Likert scale)					
Very dissatisfied	11 (24.4)	6 (28.6)	4 (25)	1 (12.5)	
Dissatisfied	6 (13.3)	3 (14.3)	1 (6.3)	2 (25)	
Neither satisfied nor dissatisfied	21 (46.7)	10 (47.6)	8 (50)	3 (37.5)	
Satisfied	6 (13.3)	2 (9.5)	3 (18.8)	1 (12.5)	
Very satisfied	1 (2.2)	0	0	1 (12.5)	
Employed Mothers	35 (77.8)	16 (76.2)	13 (81.3)	6 (75.0)	0.915
Depressive Symptomatology					
PHQ-9 score ≥ 10	9 (20.0)	5 (23.8)	4 (25.0)	-	0.295
Eating disorders symptomatology					
EAT-26 score ≥ 20	7 (15.6)	3 (14.3)	2 (12.5)	2 (25.0)	0.711
Feeding practices (CFPQ)					
(5-point Likert scale)					
Monitoring	4.11 (0.77)	4.15 (0.84)	4.09 (0.71))	4.00 (0.79)	0.762
Child Control	2.91 (0.67)	2.81 (0.79)	3.10 (0.54)	2.78 (0.55)	0.413
Emotional Regulation/Food as Reward	2.05 (0.73)	2.02 (0.73)	2.01 (0.72)	2.21 (0.84)	0.852
Healthy Eating Guidance	4.27 (0.49)	4.25 (0.45)	4.15 (0.58)	4.54 (0.35)	0.216
Restriction	3.65 (0.69)	3.63 (0.73)	3.60 (0.74)	3.80 (0.55)	0.817
Pressure to eat	1.35 (0.50)	1.29 (0.41)	1.42 (0.45)	1.37 (0.80)	0.497
SDQ total score group					0.385
(parental version)					
Clinical	4 (8.9)	2 (9.5)	1 (6.3)	1 (12.5)	
Borderline	7 (15.6)	2 (9.5)	2 (12.5)	3 (37.5)	
Normal	34 (75.6)	17 (81.0)	13 (81.3)	4 (50.0)	

Values are expressed as mean (SD) for continuous variables and as absolute numbers (n) and frequencies (%) for categorical variables. ΒΜΙ: Body Mass Index, PHQ-9: Patient Health Questionnaire—9, EAT-26: Eating Attitudes Test—26, CFPQ: Comprehensive Feeding Practices Questionnaire, SDQ: Strengths and Difficulties Questionnaire. * Pearson Chi-square, test, One-Way ANOVA, or Kruskal−Wallis as indicated.

**Table 4:** Changes in cardiometabolic parameters of children according to BMI z-score group.

		Total		Group 1 Decrease in BMI z-Score ≥0.2		Group 2 Decrease in BMI z-Score ≥0.0 <−0.2		Group 3 Increase in BMI z-Score >0.00–0.25	* *p*-Value for Differences between Groups
	n		n		n		n		
BMI z-score	45	−0.21 (0.26)	21	−0.43 (0.20)	16	−0.08 (0.06)	8	0.12 (0.09)	**<0.001**
Waist-to-height ratio	44	−0.001 (0.04)	20	−0.02 (0.03)	16	0.01 (0.04)	8	0.02 (0.02)	**0.003**
Systolic BP (mmHg)	44	−1.05 (12.25)	20	0.45 (13.10)	16	−4.44 (13.38)	8	2.00 (5.37)	0.373
Diastolic BP (mmHg)	44	−0.50 (6.67)	20	−1.65 (9.02)	16	−0.25 (7.07)	8	1.88 (4.79)	0.550
Glucose (mg/dL)	44	−0.80 (7.73)	21	−2.76 (8.61)	16	4.44 (6.51)	7	0.0001 (6.9)	0.256
Insulin (μUI/mL)	43	−2.04 (10.99)	20	−4.86 (13.05)	15	−1.21 (8.55)	8	3.46 (7.63)	0.162
HOMA-IR	42	−0.50 (2.83)	20	−1.27 (3.46)	15	−0.13 (2.03)	7	0.94 (1.53)	0.133
HbA1c (%)	42	**−0.10 (0.24) ^1^**	20	−0.05 (0.22)	14	**−0.24** **(0.27) ^1^**	8	0.001 (0.16)	**0.026**
Total cholesterol (mg/dL)	44	0.38 (15.29)	20	−4.43 (12.10)	16	−0.63 (12.07)	8	14.38 (19.32)	**0.009**
LDL cholesterol (mg/dL)	43	0.44 (12.01)	19	−4.63 (10.71)	16	0.81 (8.58)	8	**11.75 (14.04) ^1^**	**0.003**
HDL chole−−sterol (mg/dL)	44	0.55 (7.39)	20	2.02 (6.85)	16	−3.06 (7.58)	8	4.13 (5.89)	0.073
Triglycerides (mg/dL)	44	−5.84 (42.33)	20	−17.75 (41.81)	16	8.69 (48.96)	8	−5.13 (15.39)	0.257
SGOT (U/L)	45	**−1.84 (5.12) ^1^**	21	−0.95 (5.91)	16	−2.88 (3.16)	8	−2.13 (6.22)	0.493
SGPT (U/L)	45	**−2.95 (9.07) ^1^**	21	−4.85 (8.89)	16	−4.25 (6.72)	8	4.63 (10.72)	0.104
Cortisol (μg/dL)	42	−1.54 (5.86)	20	**−3.87 (6.34) ^1^**	15	0.06 (5.10)	7	1.66 (3.25)	**0.037**
ACTH (pg/mL)	38	−1.37 (32.25)	17	**−11.23 (21.87) ^1^**	14	11.04 (44.61)	7	−2.23 (13.85)	0.317

Values are expressed as mean (SD). HOMA IR: Homeostatic Model Assessment for Insulin Resistance, HbA1c: Glycated hemoglobin, LDL: LDL-cholesterol: Low-density lipoprotein cholesterol, HDL: High-density lipoprotein cholesterol, SGPT: Serum Glutamic Pyruvic Transaminase, SGOT: Serum Glutamyl Oxaloacetic Transaminase, ACTH: Adrenocorticotropic hormone. * One-Way ANOVA or Kruskal−Wallis as indicated. ^1^ *p* value within group < 0.05 (paired sample *t*-test or Wilcoxon test as indicated). Statistically significant differences are presented in bold.

**Table 5:** Multivariate regression analysis results between BMI z-score change and HOMA—IR change.

Independent Variables	Dependent Variable: HOMA-IR Change (μUI/mL) (n = 42)				
	Beta-Coefficient	Standard Error	95% Confidence Interval		* *p*-Value
BMI z-score change	3.60	1.74	0.07	7.14	**0.046**
Baseline pubertal stage	0.50	1.27	−2.08	3.08	0.697
Sex	−1.82	1.13	−4.12	0.47	0.116
Baseline BMI z-score	−0.16	0.32	−0.81	0.48	0.610
Baseline age (years)	−0.24	0.37	−0.99	0.50	0.512

* Statistically significant differences are presented in bold.

**Table 6:** Multivariate regression analysis results between BMI z-score change and SGPT change.

Independent Variables	Dependent Variable: SGPT Change (U/L) (n = 45)				
	Beta-Coefficient	Standard Error	95% Confidence Interval		* *p*-Value
BMI z-score change	11.90	5.52	0.74	23.06	**0.037**
Baseline pubertal stage	−0.46	3.91	−8.37	7.46	0.907
Sex	2.93	3.36	−3.87	9.74	0.388
Baseline BMI z-score	0.91	1.02	−1.15	2.98	0.375
Age at baseline (years)	0.58	1.06	−1.56	2.72	0.587

* Statistically significant differences are presented in bold.

**Table 7:** Multivariate regression analysis results between BMI z-score change and cortisol change.

Independent Variables	Dependent Variable: Cortisol Change (μg/dL) (n = 42)				
	Beta-Coefficient	Standard Error	95% Confidence Interval		* *p*-Value
BMI z-score change	9.96	3.58	2.71	17.22	**0.008**
Baseline pubertal stage	−4.35	2.63	−9.68	0.98	0.106
Sex	1.33	2.15	−3.04	5.70	0.542
Baseline BMI z-score	−0.32	0.64	−1.61	0.97	0.622
Age at baseline (years)	0.59	0.69	−0.82	1.99	0.402

* Statistically significant differences are presented in bold.

**Table 8:** Changes to food parenting practices according to BMI z-score change.

		Total		Group 1 Decrease in BMI z-Score ≥0.2		Group 2 Decrease in BMI z-Score ≥0.0 <−0.2		Group 3 Increase in BMI z-Score >0.00–0.25	* *p*-Value for Differences between Groups
	n		n		n		n		
Healthy eating guidance	42	**0.11 (0.38) ^1^**	19	0.23 (0.41)	15	0.14 (0.30)	8	−0.21 (0.29)	**0.013**
Monitoring	42	0.14 (0.69)	19	0.18 (0.36)	15	0.13 (0.72)	8	0.06 (0.56)	0.845
Child control	42	−0.12 (0.59)	19	−0.21 (0.58)	15	−0.24 (0.62)	8	0.33 (0.37)	**0.037**
Emotional feeding/Food as reward	42	**−0.21 (0.48) ^2^**	19	−0.35 (0.48)	15	−0.10 (0.46)	8	−0.10 (0.53)	0.257
Restriction	42	−0.13 (0.55)	19	−0.34 (0.65)	15	0.01 (0.31)	8	−0.40 (0.61)	0.169
Pressure to eat	42	0.16 (0.74)	19	0.19 (0.87)	15	0.20 (0.75)	8	0.04 (0.33)	0.953

Values are expressed as mean (SD), the scale that was used to define food parenting practices is a 5-point Likert scale. * Kruskal−Wallis for differences between groups. ^1^ *p* value = 0.051 (within group). ^2^ *p* value = 0.007, (within group). Statistically significant differences are presented in bold.

**Table 9:** Multivariate regression analysis results between healthy eating guidance change and BMI z-score change.

Independent Variables	Dependent Variable: BMI z-Score Change (n = 42)				
	Beta-Coefficient	Standard Error	95% Confidence Interval		* *p*-Value
Healthy eating guidance change	−0.29	0.10	−0.49	−0.08	**0.007**
Maternal education	0.05	0.06	−0.08	0.18	0.40
Maternal marital status	−0.07	0.09	−0.26	0.12	0.489

* Statistically significant differences are presented in bold.

## Data Availability

The data presented in this study are available on request from the corresponding author. The data are not publicly available due to privacy restrictions.
